# Purification, characterization, and antitumor activity of a novel glucan from the fruiting bodies of *Coriolus Versicolor*

**DOI:** 10.1371/journal.pone.0171270

**Published:** 2017-02-08

**Authors:** Annoor Awadasseid, Jie Hou, Yaser Gamallat, Shang Xueqi, Kuugbee D. Eugene, Ahmed Musa Hago, Djibril Bamba, Abdo Meyiah, Chiwala Gift, Yi Xin

**Affiliations:** 1 Department of Biotechnology, Dalian Medical University, Dalian, P.R. China; 2 Department of Biochemistry and Molecular Biology, Northeast Normal University, Changchun, P.R. China; 3 Department of Biochemistry & Food Sciences, University of Kordofan, El-Obeid, The Republic of Sudan; 4 Department of pathology and pathophysiology, Dalian Medical University, Dalian, P.R. China; Centro Cardiologico Monzino, ITALY

## Abstract

Cancer is one of the most common causes of deaths worldwide. Herein, we report an efficient natural anticancer glucan (CVG) extracted from *Coriolus Versicolar* (CV). CVG was extracted by the hot water extraction method followed by ethanol precipitation and purified using gas exclusion chromatography. Structural analysis revealed that CVG has a linear α-glucan chain composed of only (1→ 6)-α-D-Glc*p*. The antitumor activity of CVG on Sarcoma-180 cells was investigated *in vitro* and *in vivo*. Mice were treated with three doses of CVG (40, 100, 200 mg/kg body weight) for 9 days. Tumor weight, relative spleen, thymus weight, and lymphocyte proliferation were studied. A significant increase (*P< 0*.*01*) in relative spleen and thymus weight and a decrease (*P< 0*.*01*) in tumor weight at the doses of 100 and 200 mg/kg were observed. The results obtained demonstrate CVG has antitumor activity towards Sarcoma-180 cells by its immunomodulation activity.

## Introduction

Cancer is among the most dangerous diseases threatening human life. Natural drugs have emerged as promising approaches for cancer due to their safety and absence of side effects compared with surgery and chemotherapy [1, 2]. Natural drugs such as Vincristine, Topotecan, and Paclitaxel extracted from *Catharanthus roseus*, *Camptotheca acuminata*, and *Taxus brevifolia* respectively have antitumor activities, inhibiting tumor growth by augmenting ConA and LPS-induced splenocyte proliferation, binding to the protein tubulin, stopping the cell chromosomes separation during metaphase and induction of apoptosis. However, these extracts have associated side effects such as muscle and joint pains, hair loss, loss of appetite, diarrhea, nausea and vomiting. Immunostimulation has been considered as one of the possible mechanisms contributing to tumor growth prevention; and is related to immunomodulatory activity through Th1, Th2, and Th17 regulatory activation, hemocytoblasts, regulatory T-cells and mesenchymal stromal cells [3, 4].

Previous studies have isolated several chemical components from CV mushroom with anticancer activities such as protein-bound polysaccharides (PBP), Polysaccharopeptide (PSP), *Coriolus versicolor* polysaccharides (CVP), D- β-1,3-D-glucans, Protein-bound polysaccharide-K (PSK), *Coriolus Versicolor* polysaccharide-B (CVPs-B), and *Coriolus Versicolor* extract (CVE). PBP could trigger the apoptosis of ER-positive MCF-7 cells partly via upregulation of the p53 protein expression [5]. PSP significantly increased the percentage of CD4+ T lymphocytes, the ratio of CD4+/CD8+/CD14+/CD16_ and the quantity and percentage of the B lymphocytes and finally enhanced the immune system of cancer patients [6, 7]. CVP could induce cell cycle arrest or slowing, apoptosi, and caspase-3 expression [8]. Several research papers have also revealed that the mechanism of D- β-1,3-D-glucans is due to their triple helix conformation as their tertiary structure [9, 10]. PSK on the other hand, can modulate the expression of major histocompatibility complex (MHC) classI, inhibit NF-<kappa>B activation, downregulate the antiapoptotic molecules cIAP-1 and leads to activation of caspase-3 resulting in apoptosis of cancer cells, induce production of interleukin 8 by reacting with circulating monocytes, and also activates CTLs and maturation of dendritic cells [11, 12]. CVPs-B can inhibit proliferation and enhance apoptosis of Eca109 cells; inhibit the expression of the osteopontin (OPN) gene; down-regulate glycosaminoglycan (GAG) expression on the surface of macrophages; affect the expression of inflammatory chemotactic factor; and enable the cells proceed rapidly to the resting phase of cell growth [13]. CVE has an ability to inhibit certain proinflammatory cytokines. The antiinflammatory activity of CVE in Inflammatory Bowel Disease (IBD) might be mediated by the inhibition of signal transducer and activator of transcription (STAT) STAT 1 and STAT 6 in response to IFN-γ and IL-4 expression [14]. All these chemical components of CV mushroom mentioned above are known to play an important role in suppressing tumor cells.

Glucans belong to a group of physiologically active compounds, known as carbohydrates, consisting of linked glucose molecules, and represent highly conserved structural components of seaweed, fungi and cell wall in yeast [15, 16]. The role of glucans as a biologically active compound has been well established. Glucans have been successfully used to treat high risk neuroblastoma [17–25]. In addition, glucans have immunomodulatory role by augmenting the amount of natural killer cells and immunoglobulins. Not all glucans reported have cholesterol lowering ability, activation of bone marrow cell production, activation of macrophages and improving resistance to cancer cells [26–28].

Herein, we report the antitumour activity of a water-soluble glucan (CVG) extracted from *Coriolus Versicolar in vitro* and *in vivo* against Sarcoma-180 cells… The structural related analysis and function of CVG were also investigated using different characterization tools.

## Results

### Extraction, purification, and molecular weight of the glucan

The High-performance gel-permeation chromatography (HPGPC) results show only a single symmetrical peak revealing the homogeneity of the obtained CVG (S1 Fig). Also, there is no observed absorption peak at 280 nm, implying absence of the protein molecules in the CVG skeletal.

**Fig 1:**
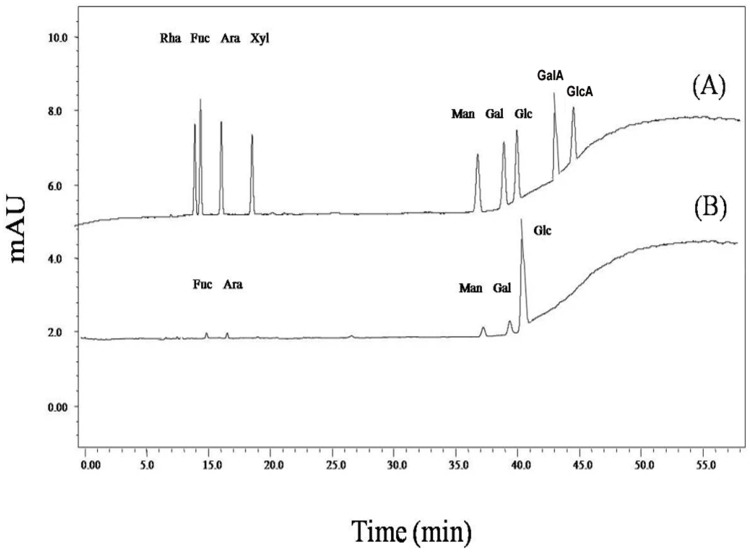
(A) and (B) represented the standard monosaccharides and a monosaccharides in the CVG (D-Fuc, D-Ara,D-Man,D-Gal and D-Glc) after hydrolyzed with TFA, respectively.

HPLC was employed to determine the molecular weight (*M*_*w*_) and monosaccharide composition of the purified CVG. The results revealed the *M*_*w*_ of CVG to be around 8.8 KDa. The carbohydrate composition of CVG analyzed consists of D-Fuc, D-Ara,D-Man, D-Gal and D-Glc, with a molar ratio of 1.0/1.1/3.0/3.9/ 350.7 respectively (Fig 1).

### NMR spectroscopy

The ^13^C NMR spectrum of CVG (S2 Fig) shows only a signal peak in the anomeric region at 102.52 ppm. This is due the presence of α-D-linked glucopyranosyl molecule in the glucan structure, also indicates that, the glucan is composed of only one sugar type in the main chain [29, 30]. The absence of signal at δ 82–88 implies that all sugar residues in the obtained glucan are in the form of pyranose. These results are in agreement with previous research reports [31]. Heteronuclear multi-bond correlation (HMBC) and heteronuclear multi-quantum coherence (HMQC) analyses contributed to the structural elucidation of the purified glucan, since the coupling of protons of the units made possible assignments of their respective carbons. The anomeric proton signal at δ 4.70 in the ^1^H NMR spectrum of CVG (S3 Fig), and coupling constant values of *J*H-1, H-2 (~ 2.9 Hz) and *J*H-1, C-1 (~ 171 Hz), indicates the sugar residues in the glucan are α-glycosidically. The resonances at 74.79, 75.65, 69.63, 72.91 and 60.73 ppm were assigned to C-2, C-3, C-4, C-5 and C-6 of glycosidic ring. This result was confirmed by HMBC and HMQC spectra (Table 1 and S4 Fig), which provided the signals corresponding to long-range connections among protons and the carbons placed at two and three bonds. Two units of glucose (A and A´) were considered. The interresidual ^1^H/^13^C cross-peaks were identified between H-1 (4.70 ppm) of residue A and C-6 (60.73 ppm) of residue A´ (A H-1/A´ C-6), C-1 (102.52 ppm) of residue A and H-6a (3.59 ppm) and H-6b (3.21 ppm) of residue A´ (A C-1/A´ H-6a; A C-1/A´ H-6b), and *vice versa*. Other cross-peaks are shown in S4 Fig. The units of α- Glc*p* residues had signals of C-1/H-1 at 102.52/4.70 assigned from HMQC. The signals at 74.79/3.86, 75.65/3.89, 69.63/3.70, 72.91/3.84 and 60.73; 59.94/3.59; 3.21 arose from C-2/H-2 to C-6/H-6 of Glc*p* units. The down fitting of the signal of C-1 indicates the presence of methyl glycoside due to α-glycosylation effect. Based on the aforementioned results, the structure of the obtained CVG is established to be

[→6)−α−D− Glcp−(1→]n

**Table 1:** ^13^C NMR and ^1^H NMR spectral assignments of (CVG).

Sugar residues	Chemical shifts, δ (ppm)					
	C-1	C-2	C-3	C-4	C-5	C-6a/6b
	H-1	H-2	H-3	H-4	H-5	H-6a/6b
→6)-α-D-Glc*p*-(1→	102.52	74.79	75.65	69.63	72.91	60.73/59.94
	4.70	3.86	3.89	3.70	3.84	3.59^a^/3.21^b^

^a, b^ Interchangeable.

### Methylation analysis

The GC-MS was used to investigate the methylated products in the extracted CVG. The results shows the methylated groups are completely converted into alditol acetates, including 1,5,6-tri-O-acetyl-2,3,4-tri-O-methyl-D-glucitol with mass fragment of 43,45,87,101,117,129,161, and 233 *m/z* (Table 2). Based on the obtained results, the α-glucan is proposed to be a linear chain containing only (1→6)-linked-D-glucopyranosyl.

**Table 2:** Methylation analysis data for (CVG).

Methylated sugar	Glycosyl linkage	Molar ratio (%)	Retention time (min)	Mass fragment (m/z)
2,3,4-Me_3_-Glc*p*	→ 6)- α-D-Glc*p*-(1→	5.1	16.913	43,45,87, 101,117,129, 161, 233

### FT-IR spectroscopy

**Fig 2:**
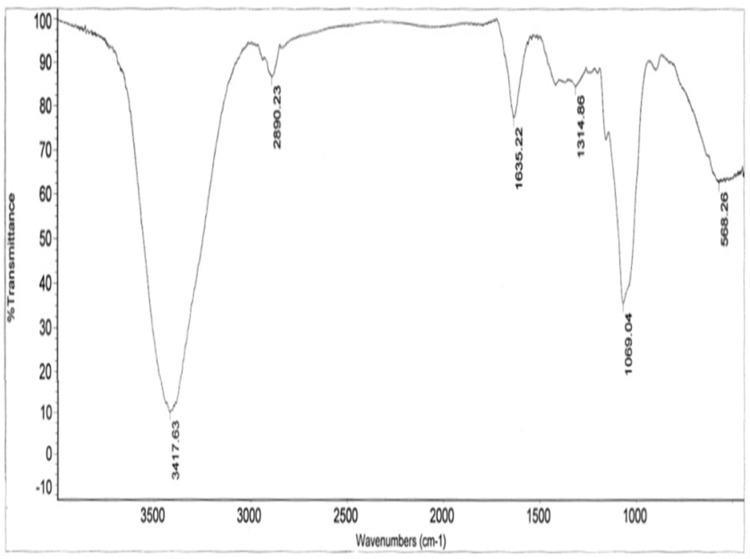
FT-IR spectrum with detector (DTGS) in a range of 4000–400 cm^−1^ of the (CVG).

Fig 2 shows the FT-IR spectrum of the CVG in the range of 4000–400 cm^−1^. The stretching vibration peaks at 3417.63, 2890.23, 1314.86, and 1069.04 cm^−1^ are assigned to the O-H, C-H, C-O, and pyranoside in conformity with previous reports [32, 33]; the peak at 568.26 cm^−1^ however, is attributed to be the α− configuration of sugar units. Notably, the lack of absorption peak at 1720 cm^−1^ indicates the absence of uronic acid in the CVG [34].

### *In vitro* antitumor activity

**Fig 3:**
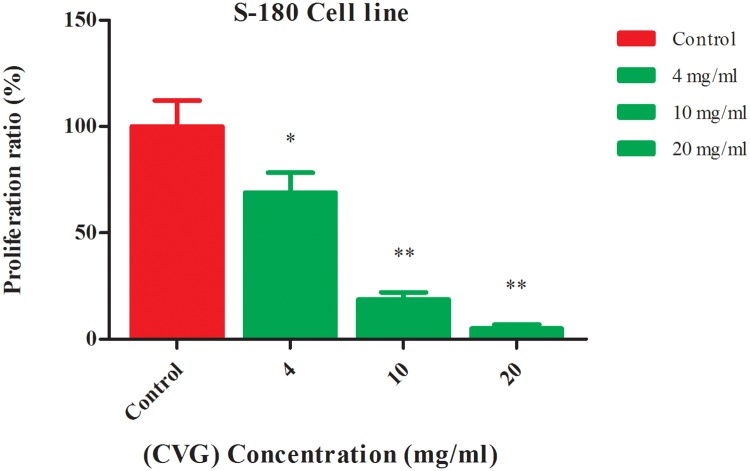
Proliferation ratio (%) of S-180 sarcoma cells at different concentrations (4, 10 and20 mg/ml) of CVG. (* P < 0.05 and ** P < 0.01 with respect to the control).

The *in vitro* antitumor activity of CVG was determined with concentrations of 4, 10, 20 mg/ml against the proliferation of S-180 cells (S1 Table). The growth inhibition of S-180 cells was dependent on the concentration of CVG (Fig 3). The growth inhibition of S-180 cells achieved about 95% at CVG 20mg/ml; and might be attributed to its high molecular weight as reported previously [35].

### *In vivo* antitumor activity

#### Tumor, spleen and thymus weight

**Fig 4:**
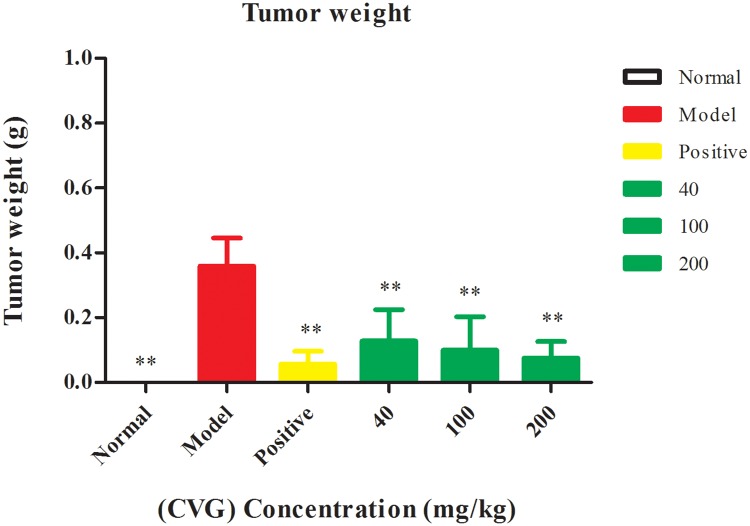
Effect of the (CVG) on tumor regression of tumor- bearing mice, The (CVG) was dissolved in normal saline and were administered i.g.; while control group received normal saline, normal control group (normal saline), positive control group (Cyclophosphamide, 20 mg/kg body weight). The dose volume was 0.2 ml. Values are means ± SD of ten mice, (** significant with respect to the model control with *P* < 0.01).

The results indicate a significant antitumor effect of CVG (Fig 4) compared with the control group (*P*< 0.01). The determined *in vivo* antitumor activities of CVG are found to be 64%, 72%, and 79% at concentrations 40, 100, and 200 mg/kg of CVG respectively. Also, CVG increased the thymus and spleen index in tumor- bearing mice (S2 Table). In comparison, the growth inhibition using 20 mg/kg of Cyclophosphamide is around 84%. Although, the antitumor activity of CVG is lower than Cyclophosphamide, it is preferred due to its safety. Specifically, Cyclophosphamide decreases the organism leukocytes and deteriorates the immune functions [36].

#### Spleen lymphocyte proliferation

**Fig 5:**
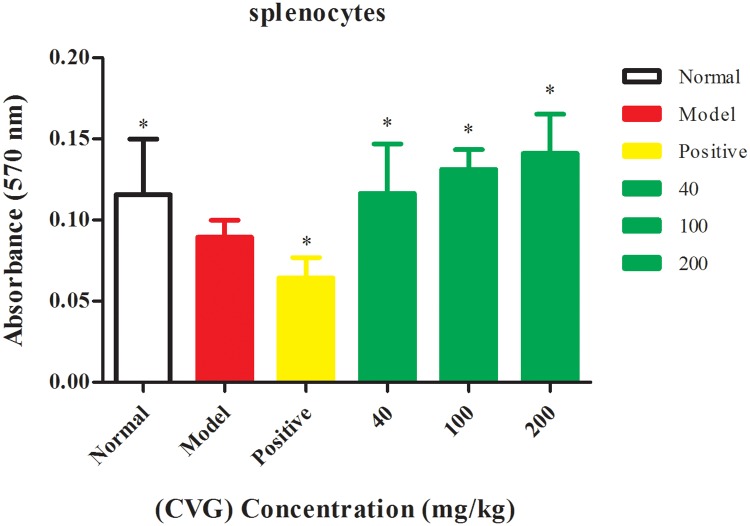
Effect of the (CVG) on splenocytes proliferation, proliferation activity was expressed as the absorption at 570 nm. Values are means ± SD of ten mice, (* significant with respect to the model control group at *P* < 0.05).

The proliferation of the splenocytes was employed to investigate the effect of CVG on the cellular immune responses (S3 Table). Fig 5 reveals an increased proliferation of splenocytes in the presence of CVG compared with the positive control (*P*< 0.05). This is attributed to the high immunomodulatory activity of CVG.

#### Effect on macrophage phagocytosis

**Fig 6:**
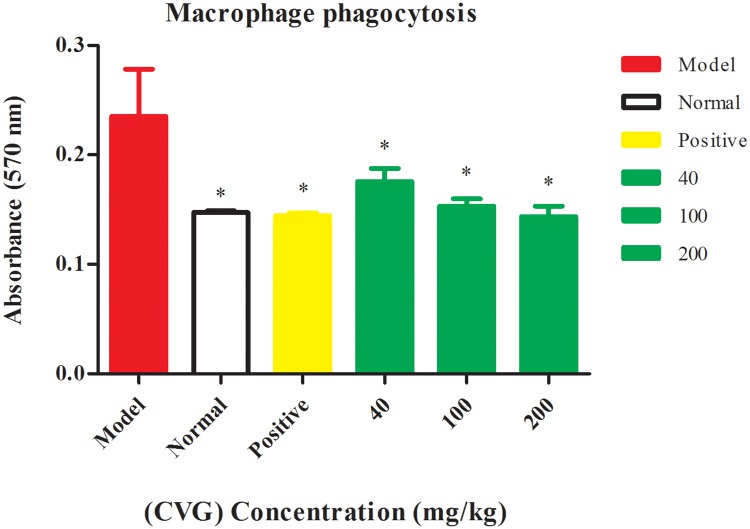
Effect of the (CVG) on phagocytosis of macrophage in the bearing tumor mice, Phagocytosis activity was expressed as the absorption at 570 nm. Values are means ± SD of ten mice, (* significant with respect to the model control at *P* < 0.05).

The immunomodulation activity of CVG was investigated using the neutral red method. S4 Table presents the effect of different concentrations of CVG on the phagocytosis of the macrophage. The phagocytosis of macrophages was significantly decreased by CVG compared with the model control (Fig 6); but slightly higher compared with the normal and positive groups. This result is in concordance with previously published reports [37, 38].

## Discussion

*Coriolus versicolor* (CV), known as Yunzhi in China, is a mushroom belonging to species of the Basidiomycetes class of fungi, which has been widely used as a magic drug to treat cancer and immune deficiency related illnesses [39, 40]. CV contains starch, fiber, chitin, and high amount of protein [26]. Besides these compounds, other polysaccharides and constituents of interest have been found in CV including the antioxidant phenolic compounds gallic acid, protocatechuic acid, and catechin; as well as calcium and minerals, vitamins B1, B2, C and D, ergosterol, selenium and eritadenine [41]. There are some differences both in structure, *M*_*W*_ and of extracted *Coriolus versicolor* polysaccharides (CVP) due to culture conditions, varied strains, and other factors. The medium component also affects the *M*_*W*_ of the polysaccharide [8]. Both products Polysaccharopeptide (PSP), and Protein-bound polysaccharide-K (PSK) have a molar mass of approximately 100 kDa [12]. The extracellular polysaccharide (EPS) contains small amounts of galactose, mannose, arabinose, xylose and predominantly glucose. The main EPS is composed of β-(1–3) and β-(1–6)-linked D-glucose molecules [5], while PSP and PSK contain α-(1–4) and β-(1–3) glucosidic linkages in their polysaccharide moieties [42]. D-glucose is the major monosaccharide present while fucose, galactose, mannose, and xylose are the other principal monosaccharides in PSK. However PSP contains arabinose and rhamnose [43, 44]. PSK and PSP are dark brown or light powders that are still stable in hot water. The compounds are tasteless, odorless and do not have a definite melting point. The PSK and PSP polymers are soluble in water but insoluble in hexane, benzene, chloroform, pyridine, and methanol. The aqueous solution of PSP (1 g/100 ml water) is neutral, with a pH value of between 6.6–7.2. The α _D_^25^ (specific rotation) value of the PSP solution is in the range of 0–30°C [9].

In the present study, we successfully isolated and purified the CVG, clarify its composition, and demonstrated that it can strengthen the immune system in the tumor- bearing mice and inhibit the growth of S- 180 directly in a dose dependent way compared with the control. This finding is evident by the observed increased glucan-induced thymus and spleen indexes in the tumor-bearing mice. In addition, augmenting CVG with ConA resulted in splenocyte proliferation, leading to stimulation of T-Cells and B-Cells. Also, CVG glucan significantly increased the white blood cell count in the tumor-bearing mice leading to engulfment and growth suppression of S-180 cells, thereby contributing to the immunity. The phagocytes, one of the earliest cell types to respond to invasion by pathogenic organisms, are key participants in the innate immune response [37]. Together with neutrophils and macrophages, they represent the first line of host defense after the epithelial barrier. They are also involved in tissue remodeling during embryogenesis, wound repair, clearance of apoptotic cells and hematopoiesis [38]. Thymus, spleen, T-Cells, B-Cells, and white blood cells can play an important role to an inactivation the oncogene, which lead to tumor regression both through a direct effect on tumor cells as well as by recruiting immune effectors that can remodel the tumor microenvironment. The judicious combination of oncogene-targeted therapy with specific immunomodulatory therapy may further increase the clinical response and long-term survival of patients [40]. Tumor formation and development are closely associated with the host immune state and hence, it is very important to improve the immunity of cancer patients [40]. The present work shows that incubation of tumor cells in glucans could suppress the growth of the tumor both *in vitro* and *in vivo*. Going on, our study seems to suggest that CVG could be a promising therapeutic agent against tumors due to its immunomodulation activity. The enhancement of host immune response is linked with inhibition of tumor growth [45–48]. The antitumor activity of CVG might be accomplished by improving host immunity. CVG is a potential tumour cell growth inhibitor and can be explored by food and paharmaceutical industries.

## Conclusions

In summary, water soluble glucan is successfully extracted from *Coriolus Versicolor*. The structural related function of CVG is characterized by using NMR, FTIR, HPLC, and methylation analysis. The results revealed that, the CVG consist of (1→ 6)-α-D-Glc*p*. The obtained CVG exhibited a significant antitumor activity against Sarcoma-180 cells in concentration dependent manner. The antitumor effect of CVG is attributed to immunomodulation activity of (1→ 6)-α-D-Glc*p*.

## Materials and methods

### Materials

Fruiting body of *Coriolus versicolor* used in this study is cultivated in Changbai Mountain district and identified by Professor Yi Xin at Department of Biotechnology, Dalian Medical University, Dalian, P.R. China. TriFlouracetic Acid (TFA) CAS No: 76-05-1, m-hydroxydiphenyl, CAS No: 580-51-8, Sulfamic Acid CAS No: 5329-14-6, Sephacryl S-500 High Resolution, CAS No: GE17-0613-01, 3-(4, 5-dimethylthiazolyl-2)-2, 5-diphenyl tetrazolium bromide (MTT) Cat No: 3511-096-K, Concanavalin A (ConA) CAS No: 11028-71-0, Neutral Red CAS No: 553-24-2, and Cyclophosphamide (CP) CAS No: 6055-19-2, were purchased from Sigma Chemical Co. (St. Louis, MO, USA). Medium RPMI 1640 with improved nutrient solution was purchased from Gibco industry (Grand Island, NY, USA, Cat No: 11875119). CCK-8, Sarcoma 180 (S-180) were purchased from (KeyGENBioTCH, Cat No: KGA317) China. All other reagents and chemicals used were of analytical grade made in China.

This study was carried out in strict accordance with the recommendations in the Guide for the Care and Use of Laboratory Animals of the National Institutes of Health. The protocol was approved by the Committee on the Ethics of Animal Experiments of the Dalian Medical University. All surgeries were performed under sodium pentobarbital anesthesia, and efforts were made to minimize suffering.

### Extraction and fractionation of polysaccharides

500g of dried fruiting bodies of *Coriolus versicolor* was soaked in 6 L of (ddH_2_O) for 24 h, followed by boiling in 80% ethanol for 15 min to remove pigments, and further washed by ddH_2_O to remove the ethanol. The fruiting bodies were soaked again in 8 L of ddH_2_O overnight and extracted three times with 0.2% Oxalic Acid at 70−80°C every 2 h. The supernatants were filtered through gauze, and precipitated to recover the water-insoluble materials by the slow addition of ethanol 95% (v/v) with stirring until the concentration of alcohol reached 75%. The mixture was then kept overnight and centrifuged at 4500 rpm for 10 min to separate the supernatant and residue (crude polysaccharides). The deproteination of the obtained crude polysaccharides was performed by using Sevage method [49].

The crude polysaccharides fraction, CVG-0 yield: 54g, was further dissolved in ddH_2_O and applied to DEAD-cellulose anion exchange chromatography column (2.6 × 30 cm), eluted with ddH_2_O in a gradient of 0−2 mol/L NaCL successively at a flow rate of 60 ml/h. The yielded fractions were combined according to the total sugar content quantified by the Phenol–Sulfuric acid method. The main peak of CVG-0 was further fractionated on a Sephacryl S-500 column (2.6 × 30 cm) eluted with NaCL (0.15 M) at a flow rate of 30 ml/h. The main fraction was collected, concentrated, dialyzed and lyophilized to get a light yellow purified designated as CVG, yield: 5.85g, 10.8% of the crude polysaccharides.

### Determination of the monosaccharide composition

The monosaccharide composition analysis was determined by HPLC performed on a Shim-pak VP-ODS column (150 × 4.6 mm i.d) with a guard column of a Shimadzu HPLC system (LC-10 ATvp pump and UV-Vis detector) and monitored by UV absorbance at 245 nm. The CVG sample (2mg), initially was methanolyzed with 2M HCl at 80°C for 16 h, and then hydrolyzed with 2 M TFA (1 mL) at 120°C for 1 h. Elution was carried out at a flow rate of 1.0 mL/min at room temperature. The hydrolysate was derivatized to be 1-phenyl-3methyl-5-pyrazolone (PMP) derivatives and subsequently analyzed by HPLC [50]. D-Gal, D-Ara, D-Fuc, D-Rha, D-Man, D-Xyl, D-Glc, D-GlcA and D-GalA were used as sugar standards.

### Determination of homogeneity and molecular weights

The homogeneity and molecular weight (M_w_) of the CVG fractions were determined by HPGPC performed on a Shimadzu LC-10 ATvp HPLC system; fitted with TSK-GEL G-3000 PWxl or TSK-GEL G-4000 PWxl column (7.8 × 30.0 cm) gel filtration column coupled with a Shimadzu RID-10A detector set at 40°C (Shimadzu, Tokyo, Japan). A sample solution (20 μL, 5mg/mL) was injected in each run and the column was eluted with 0.2 M NaCl at a flow rate of 0.6 mL/min and 0.5 mL/min for TSK-GEL G-3000 PWxl and TSK-GEL G-4000 PWxl respectively. The data was analyzed with Millennium 32 (Waters Alliance) software. The columns were calibrated with standard dextrans within the range of M_w_ from 1000 to 100,000.

### Methylation analysis

The methylation of CVG was carried out three times using Ciucanu and Kerek method. The pre-methylated product was hydrolyzed with 85% ethanol for 4 h at 100°C, and further with 2M TFA for 6 h at 100°C. The mixture was then reduced with NaBH_4_ and acetylated with acetic anhydride and pyridine. The resulting products were analyzed by GC-MS using a Shimadzu GC-14C instrument equipped with a hydrogen flame ionization detector on Rtx-2330 column (0.32 mm × 15 mm i.d., 0.2 μm), at a temperature program of 175°C followed by 8−240°C/1 min and 8−265°C/17 min. The quantification for molar ratio was estimated using the peak areas and response factors [51, 52].

### NMR and FT-IR spectroscopies

^1^H and ^13^C NMR spectra of glucan were recorded at 27°C on a Bruker 5 mm broadband, with a spectrometer (Bruker Avance 600 MHz (German)), which operated at 126 MHz for ^13^C NMR and 600 MHz for ^1^H NMR. The sample (20 mg) was dissolved in D_2_O (99.8%, 0.5 mL), lyophilized and re-dissolved again in D_2_O (0.5 mL). The sample was finally centrifuged to remove excessive un-dissolved sample before analysis. All the experiments were recorded using standard Bruker software. FT-IR spectra were recorded on a Nicolet 6700 Thermo Scientific FT-IR spectrometer (USA) with detector (DTGS) in a range of 4000–400 cm^−1^. The sample measured on KBr discs as a film.

### Biological activity

#### *In vitro* cell proliferation assay

The *in vitro* cell proliferation assay was conducted using tetrazolium WST-8 dye (CCK-8), according to manufacturer’s instruction (KeygenDojindo, Kumamoto, Japan). S-180 sarcoma cells were grown in RPMI 1640 medium supplemented with 10% calf serum and 100 IU/mL penicillin and streptomycin under an atmosphere of 5% CO_2_ at 37°C for 72 h. Briefly, 1×10^4^ cells of (S-180) were seeded in a 96-well plate (Costa) at 37°C in 5% CO_2_ incubator and allowed to adhered for 24 h. Subsequently, cells were treated with the CVG in different concentration (4, 10, 20 mg/mL) for 72 h. The set of adhered control cells were treated similarly but without CVG in the media. The media were discarded and 10 μL/well of CCK-8 solution was added, followed by incubation for 90 min at 37°C. Finally, the absorbance was determined at 450 nm using microplate reader (Bio-Rad). All the experiments were performed in triplicate and results were expressed as the proliferation ratio (Φ) of tumor cells calculated as follows:

Φ = [(C−Cs)/C] × 100%

Where C and Cs are the average number of the viable tumor cells for the control and samples respectively.

### *In vivo* antitumor assay

#### Animals and treatment

Specific Pathogen Free (SPF) Kunming mice (18−20g, 6−7 week’s old female/ C57BL) were obtained from Animal Center of Dalian Medical University, China. They were randomized and housed 6/cage (Sixty mice) in polycarbonate cages containing sawdust bedding. The mice were housed under normal laboratory conditions, i.e., room temperature, 12/12-h light–dark cycle, with free access to standard rodent chow and water *ad libitum*. The mice were divided into six groups, each group containing 10 mice. S-180 sarcoma cells (0.2 mL, 2×10^6^ cells) were inoculated subcutaneously into right axilla of each mouse, while one group served as normal control. The mice were treated as follows: normal control group (normal saline); model (negative) control group (normal saline); positive control group (Cyclophosphamide, 20 mg/kg body weight); and three groups injected by (40, 100, 200 mg/kg body weight) of the CVG. The CVG was dissolved in normal saline, and all the groups were administered by intraperitoneal injection in a volume of 0.2 mL every day for 9 days, starting 24 h after tumor transplanting.

#### Tumor, spleen and thymus weight

After 9 days of intraperitoneal injection, mice were sacrificed by cervical dislocation. Tumor, thymus, and spleen weights of the mice were measured [53]. The antitumor activity *in vivo* of the tested samples were expressed as an inhibition ratio (%) calculated as **[(C-T)/C] × 100%**, where C and T are the average tumor weight of the model control and treated group, respectively.

#### Spleen lymphocyte proliferation assay

The spleen lymphocytes were seeded into 96-well flat-bottom microplate at 2×10^6^ cells/mL and cultured with RPMI 1640 medium, consisting of 100 μg/mL penicillin, 10% newborn bovine serum (NBS), 5 μg/mL concanavalin A (Con A), and 100 UI streptomycin. The plates incubated at 37°C in a humidified atmosphere with 5% CO_2_. After 72 h, 20 μL of MTT (5 mg/mL) was added to every well of culture plate and further incubated for 4 h at 37°C. After aspirating the supernatant from the wells, 100 μL of acidified isopropylalcohol was added and oscillated for 10 min to dissolve the colored material, and the optical density of each well was then measured at absorbance of 570 nm Bio-Rad (Hercules, CA, USA) [54].

#### Macrophage phagocytosis assay

Macrophages were prepared from Kunming mice as described previously [55]. Phagocytosis of macrophages was measured by neutral red uptake method as described previously [56, 57]. The mice were soaked in 70% ethanol after removing the eyeballs. 5 mL sterile PBS solution was injected into the peritoneal cavity of the mice, followed by kneading for 1 min, and peritoneal syphoned with a syringe and centrifuged at 2000 rpm/10 min. The erythrocytes were lysed with Tris-NH_4_CL and the cells washed three times and re-suspended in RPMI-1640 medium with 10% FBS at 2×106 cells/mL. The cell suspension (100 μL) was added in each well of 96-well plate, followed by incubation for 3 h to allow the cells to attach to the plate bottom. The supernatant was then discarded, and the wells washed with sterile PBS solution to remove non-adherent cells. 0.1% neutral red dye was added to each well (100 μL/well) and the plates incubated for 1 h at 37°C in 5% CO_2_ followed by three times wash with sterile PBS solution. Finally, 200 μL of lysis solution (acetic acid: anhydrous ethanol, 1:1) was added into each well. The mixtures were gently oscillated and then placed in a 4°C overnight. The optical density at 570 nm was measured using Bio-Rad microplate reader.

### Statistical analysis

All experiments were conducted in triplicate. Data is presented as mean ± standard deviation (SD). Statistical analysis was performed with SPSS version 17.0 software and GraphPad Prism version 5.0 software. One-way analysis of variance (ANOVA) test was used to make a statistical comparison between the treatment and the control groups. The differences were considered significant at *p< 0.05 and **p < 0.01.

## Supporting information

S1 Fig Elution profile of CVG on Sephacryl S- 500HR. The column was eluted with 0.15 M NaCL at flow rate of 0.5/min. (Vo = Void volume; Vt = Total volume).
(DOCX)

S2 Fig ^13^C NMR spectrum (125 MHz, D_2_O, 27°C) of (CVG). (DOCX)

S3 Fig ^1^H NMR spectrum (600 MHz, D_2_O, 27°C) of (CVG). (DOCX)

S4 Fig HMBC (A) and HMQC (B) spectra of CVG. (DOCX)

S1 Table The results of the *in vitro* cell proliferation of S-180 at different concentrations of the CVG. (DOCX)

S2 Table Effect of the CVG on thymus index and spleen index of tumor- bearing mice. (DOCX)

S3 Table Effect of CVG on the proliferation of splenocytes *in vivo*. (DOCX)

S4 Table Effect of the CVG on phagocytosis of macrophage *in vivo*. (DOCX)
