# Supplementary material for: Purification, characterization, and antitumor activity of a novel glucan from the fruiting bodies of Coriolus Versicolor
Source: PLoS One. 2017 Feb 8;12(2):e0171270. doi: 10.1371/journal.pone.0171270 (PMC5298263; doi:10.1371/journal.pone.0171270)
Supplement: S1 Table — (DOCX) [file pone.0171270.s005.docx]

**PLOS ONE**

**Supporting Information. PONE-D-16-40193**

Purification, Characterization, and Antitumor Activity of a Novel Glucan from the Fruiting Bodies of *Coriolus Versicolor*

Running Title: Antitumor Activity of *Coriolus Versicolor* Glucan (CVG)

Annoor Awadasseid^1, 2, 3^, Jie Hou^1^, Yaser Gamallat^1^, Shang Xueqi^1^, Kuugbee.D.Eugene^1^, Ahmed Musa Hago^4^, Djibril Bamba^1^, Abdo Meyiah^1^, Chiwala Gift^1^ and Yi Xin^1,*^

^1^ Department of Biotechnology, Dalian Medical University, Dalian 116044, P.R. China.

^2^ Department of Biochemistry and Molecular Biology, Northeast Normal University, Changchun 130024,

P.R. China.

^3^ Department of Biochemistry & Food Sciences, University of Kordofan, El-Obeid 51111, The Republic of

Sudan.

^4^ Department of pathology and pathophysiology, Dalian Medical University, Dalian 116044, P.R. China.

***** Corresponding author: Yi Xin, Department of Biotechnology, Dalian Medical University, 9 West Section, Lvshun South Road, Dalian 116044, Liaoning Province, P.R. China. E-mail: [jimxin@hotmail.com](mailto:jimxin@hotmail.com); Tel.: +86-411-8611-0295.

**S1 Table**

| **Sample** | **Cell line** | **Proliferation ratio (%)** | | | |
| --- | --- | --- | --- | --- | --- |
|  |  | **Control** | **4mg/ml** | **10mg/ml** | **20mg/ml** |
| CVG | S-180 | 100 ± 27.39 | 68.92 ± 20.90^*^ | 18.67 ± 7.455^**^ | 5.083 ± 4.165^**^ |

Values are means ± S.D. ^*, **^ Compared with control group, *P*< 0.05 and *P*< 0.01, respectively.

Supporting Information Caption

**S1 Table.** The results of the *in vitro* cell proliferation of S-180 at different concentrations of the CVG.
