# Supplementary material for: Purification, characterization, and antitumor activity of a novel glucan from the fruiting bodies of Coriolus Versicolor
Source: PLoS One. 2017 Feb 8;12(2):e0171270. doi: 10.1371/journal.pone.0171270 (PMC5298263; doi:10.1371/journal.pone.0171270)
Supplement: S2 Table — (DOCX) [file pone.0171270.s006.docx]

**PLOS ONE**

**Supporting Information. PONE-D-16-40193**

Purification, Characterization, and Antitumor Activity of a Novel Glucan from the Fruiting Bodies of *Coriolus Versicolor*

Running Title: Antitumor Activity of *Coriolus Versicolor* Glucan (CVG)

Annoor Awadasseid^1, 2, 3^, Jie Hou^1^, Yaser Gamallat^1^, Shang Xueqi^1^, Kuugbee.D.Eugene^1^, Ahmed Musa Hago^4^, Djibril Bamba^1^, Abdo Meyiah^1^, Chiwala Gift^1^ and Yi Xin^1,*^

^1^ Department of Biotechnology, Dalian Medical University, Dalian 116044, P.R. China.

^2^ Department of Biochemistry and Molecular Biology, Northeast Normal University, Changchun 130024,

P.R. China.

^3^ Department of Biochemistry & Food Sciences, University of Kordofan, El-Obeid 51111, The Republic of

Sudan.

^4^ Department of pathology and pathophysiology, Dalian Medical University, Dalian 116044, P.R. China.

***** Corresponding author: Yi Xin, Department of Biotechnology, Dalian Medical University, 9 West Section, Lvshun South Road, Dalian 116044, Liaoning Province, P.R. China. E-mail: [jimxin@hotmail.com](mailto:jimxin@hotmail.com); Tel.: +86-411-8611-0295.

**S2 Table**

| Group | Dose  (mg/kg) | Thymus index (mg/g) | Spleen index (mg/g) |
| --- | --- | --- | --- |
| Normal | ─ | 3.00 ± 0.43^**^ | 4.31 ± 1.01^**^ |
| Model | ─ | 2.03 ± 1.33 | 3.32 ± 1.62 |
| Positive | 20 | 2.94 ± 0.79^**^ | 3.68 ± 0.76^**^ |
| CVG | 40 | 3.15 ± 0.81^**^ | 4.75 ± 0.72^**^ |
| CVG | 100 | 3.45 ± 0.52^**^ | 5.18 ± 0.85^**^ |
| CVG | 200 | 3.93 ± 0.87^**^ | 5.93 ± 1.12^**^ |

Values are means ±S.D.  ^**^ Compared with model group, *P*< 0.01.

Supporting Information Caption

**S2 Table.** Effect of the CVG on thymus index and spleen index of tumor- bearing mice.
