# Supplementary material for: Purification, characterization, and antitumor activity of a novel glucan from the fruiting bodies of Coriolus Versicolor
Source: PLoS One. 2017 Feb 8;12(2):e0171270. doi: 10.1371/journal.pone.0171270 (PMC5298263; doi:10.1371/journal.pone.0171270)
Supplement: S4 Table — (DOCX) [file pone.0171270.s008.docx]

**PLOS ONE**

**Supporting Information. PONE-D-16-40193**

Purification, Characterization, and Antitumor Activity of a Novel Glucan from the Fruiting Bodies of *Coriolus Versicolor*

Running Title: Antitumor Activity of *Coriolus Versicolor* Glucan (CVG)

Annoor Awadasseid^1, 2, 3^, Jie Hou^1^, Yaser Gamallat^1^, Shang Xueqi^1^, Kuugbee.D.Eugene^1^, Ahmed Musa Hago^4^, Djibril Bamba^1^, Abdo Meyiah^1^, Chiwala Gift^1^ and Yi Xin^1,*^

^1^ Department of Biotechnology, Dalian Medical University, Dalian 116044, P.R. China.

^2^ Department of Biochemistry and Molecular Biology, Northeast Normal University, Changchun 130024,

P.R. China.

^3^ Department of Biochemistry & Food Sciences, University of Kordofan, El-Obeid 51111, The Republic of

Sudan.

^4^ Department of pathology and pathophysiology, Dalian Medical University, Dalian 116044, P.R. China.

***** Corresponding author: Yi Xin, Department of Biotechnology, Dalian Medical University, 9 West Section, Lvshun South Road, Dalian 116044, Liaoning Province, P.R. China. E-mail: [jimxin@hotmail.com](mailto:jimxin@hotmail.com); Tel.: +86-411-8611-0295.

**S4 Table**

| Group | Dose  (mg/kg) | Phagocytosis of Macrophage (A 570 nm) |
| --- | --- | --- |
| Model | ─ | 0.2350 ± 0.04328 |
| Normal | ─ | 0.1473 ± 0.001528^*^ |
| Positive | 20 | 0.1443 ± 0.002517^*^ |
| CVG | 40 | 0.1757 ± 0.01193^*^ |
| CVG | 100 | 0.1530 ± 0.006928^*^ |
| CVG | 200 | 0.1437 ± 0.009452^*^ |

Values are means ±S.D. ^*^ significant compared with model control group at *P*< 0.05.

Supporting Information Caption

**S4 Table.** Effect of the CVG on phagocytosis of macrophage *in vivo*.
